# A Rare Iatrogenic Trio: Pneumopericardium, Pneumoperitoneum, and Pericarditis

**DOI:** 10.1016/j.jaccas.2021.07.024

**Published:** 2021-10-06

**Authors:** Bharadwaj Satyavolu, Hamza A. Lodhi, Adithya Mathews, Priya Bansal, Haider Altaii, Ramez Morcos, Anand Desai, Brijeshwar Maini, Houman Khalili

**Affiliations:** a Department of Internal Medicine, Charles E. Schmidt College of Medicine, Florida Atlantic University, Boca Raton, Florida, USA; b Department of Internal Medicine, Delray Medical Center, Delray Beach, Florida, USA

**Keywords:** complication, pericardial effusion, tamponade, CT, computed tomography, *E coli*, *Escherichia coli*

## Abstract

We present a rare case of iatrogenic pneumopericardium, pneumoperitoneum, and *Escherichia coli* pericarditis after emergency pericardiocentesis for pericardial tamponade. The patient had profound bowel distention at the time of the procedure that led to iatrogenic pericardioperitoneal fistula formation along with transverse colon perforation, which manifested later after pericardial drain removal. This condition required repeat pericardiocentesis, laparoscopic colon repair, a long course of antibiotics, and an eventual pericardial window. (**Level of Difficulty: Intermediate.**)

## Central Illustration

**Figure:**
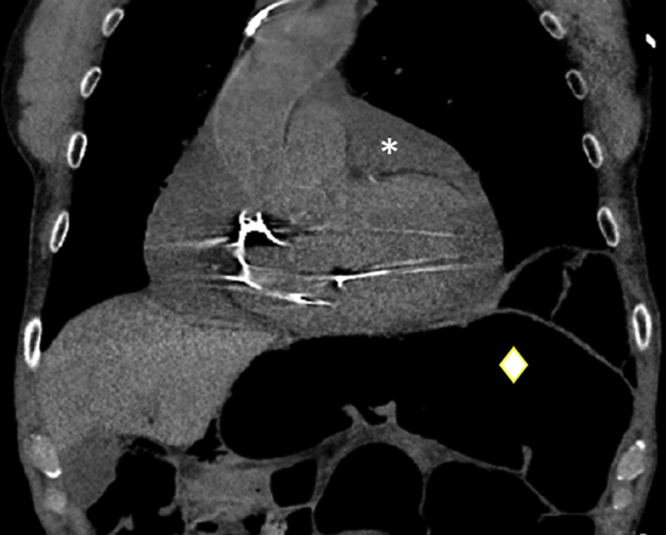

## History of Presentation

An 86-year-old, well-nourished man presented with an episode of syncope without an obvious prodrome. The patient also reported a history of chronic constipation and a possible gastrointestinal motility disorder. His blood pressure was 91/44 mm Hg, and his heart rate was 60 beat/min. His physical examination was significant for elevated jugular venous pressure and muffled heart sounds. His electrocardiogram revealed a paced rhythm with low-voltage QRS complexes. His initial laboratory test results were normal.

**Learning Objectives**

• To learn to identify and accurately diagnose pneumopericardium and/or pneumoperitoneum.
• To understand appropriate management for various presentations of pneumopericardium and possible associated complications.
• To review all available imaging modalities to determine the best approach for pericardiocentesis.

## Past Medical History

The patient’s medical history included nonischemic cardiomyopathy with cardiac resynchronization therapy with defibrillator, chronic constipation, hyperlipidemia, and carotid artery stenosis.

## Differential Diagnosis

The differential diagnosis included pericardial tamponade, cardiogenic shock, and large pulmonary embolism with right ventricular failure.

## Investigations

**Figure 1:**
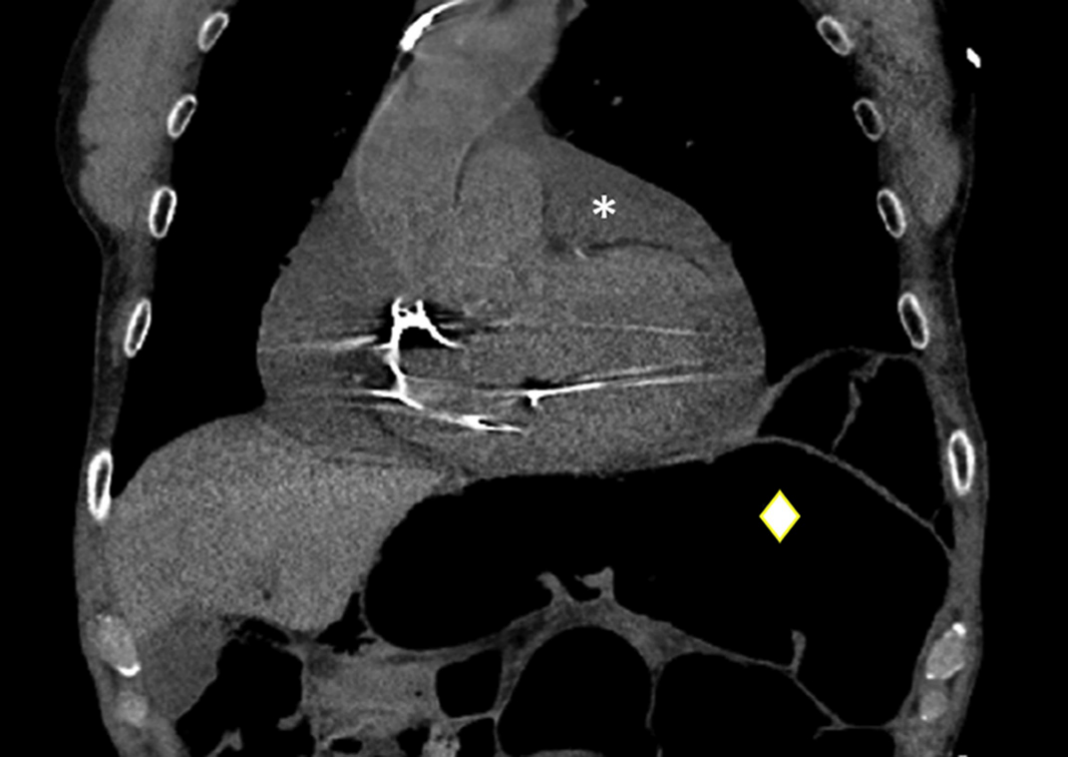
CT Imaging Computed tomography (CT) scan showing evidence of a large pericardial effusion **(asterisk)** and a distended colon **(diamond).**

**Figure 2:**
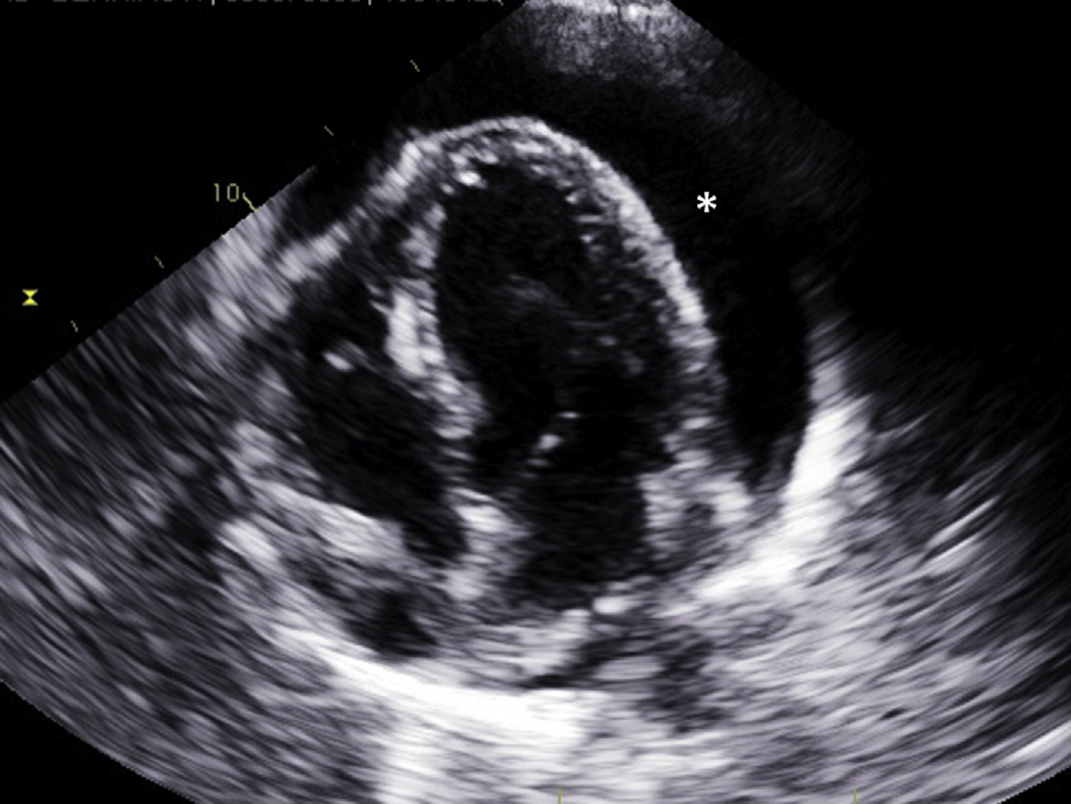
Echocardiography Transthoracic echocardiogram showing pericardial tamponade **(asterisk)**.

A chest radiograph showed an enlarged cardiac silhouette. Subsequent chest computed tomography (CT) without contrast enhancement revealed a large, circumferential pericardial effusion (Figure 1). A large pericardial effusion with tamponade physiology was confirmed by a transthoracic echocardiogram (Figure 2). After obtaining informed consent, the patient was taken to the cardiac catheterization laboratory for emergency pericardiocentesis.

## Management

**Figure 3:**
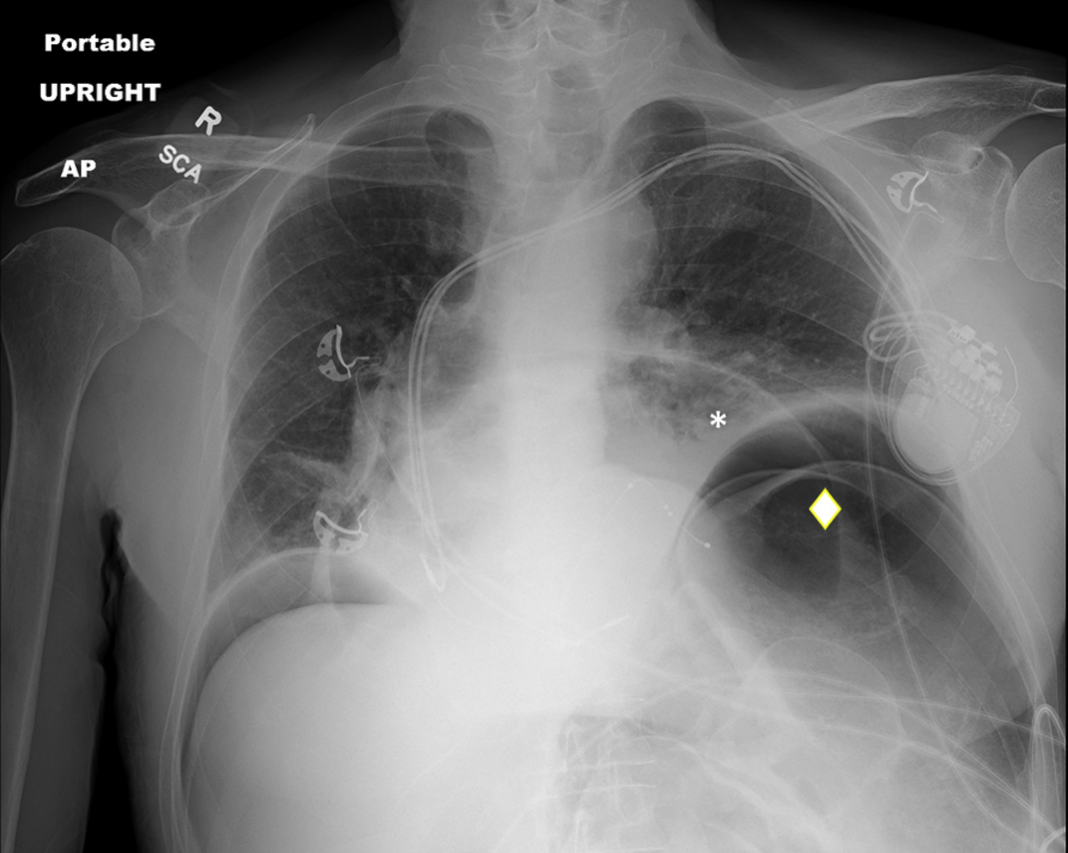
Chest Radiography Chest radiograph showing a large pneumopericardium **(asterisk)** and pneumoperitoneum **(diamond).** AP = anteroposterior; R = right; SCA = subclavian artery.

**Figure 4:**
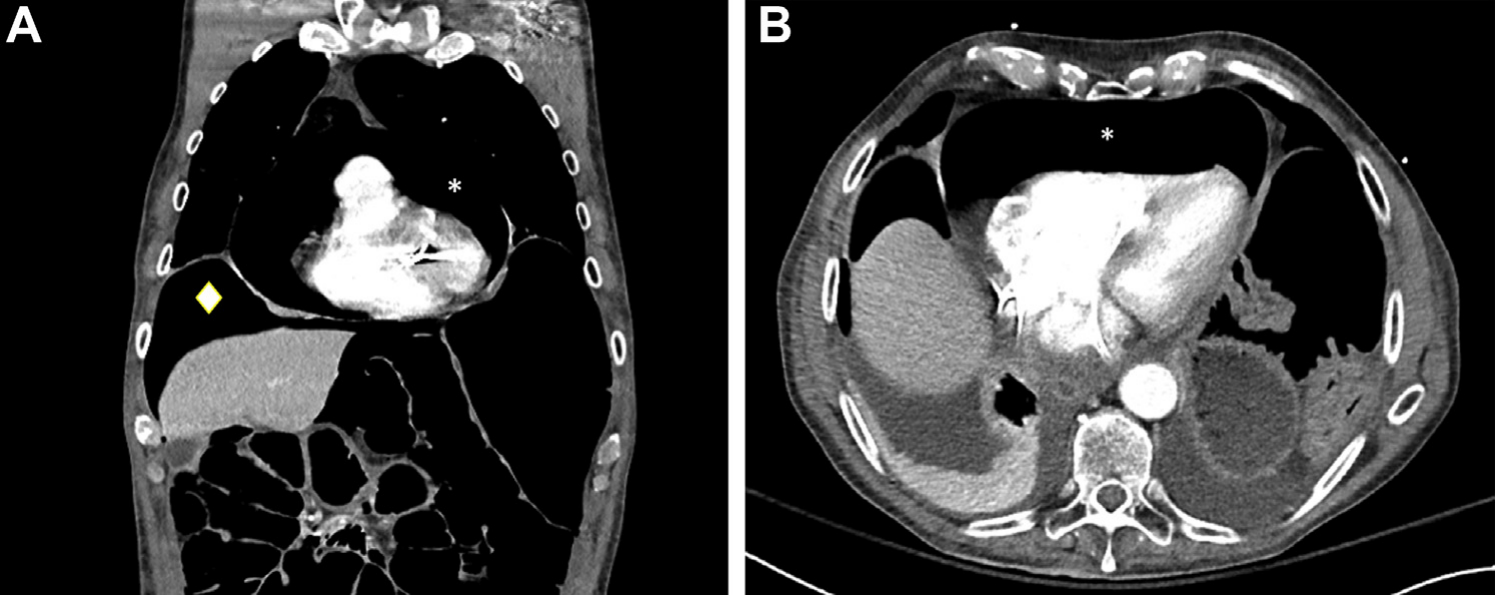
Further CT Imaging **(A)** Computed tomography (CT) scan showing a large pneumopericardium **(asterisk)** and pneumoperitoneum **(diamond). (B)** Computed tomography scan showing large pneumopericardium **(asterisk).**

**Figure 5:**
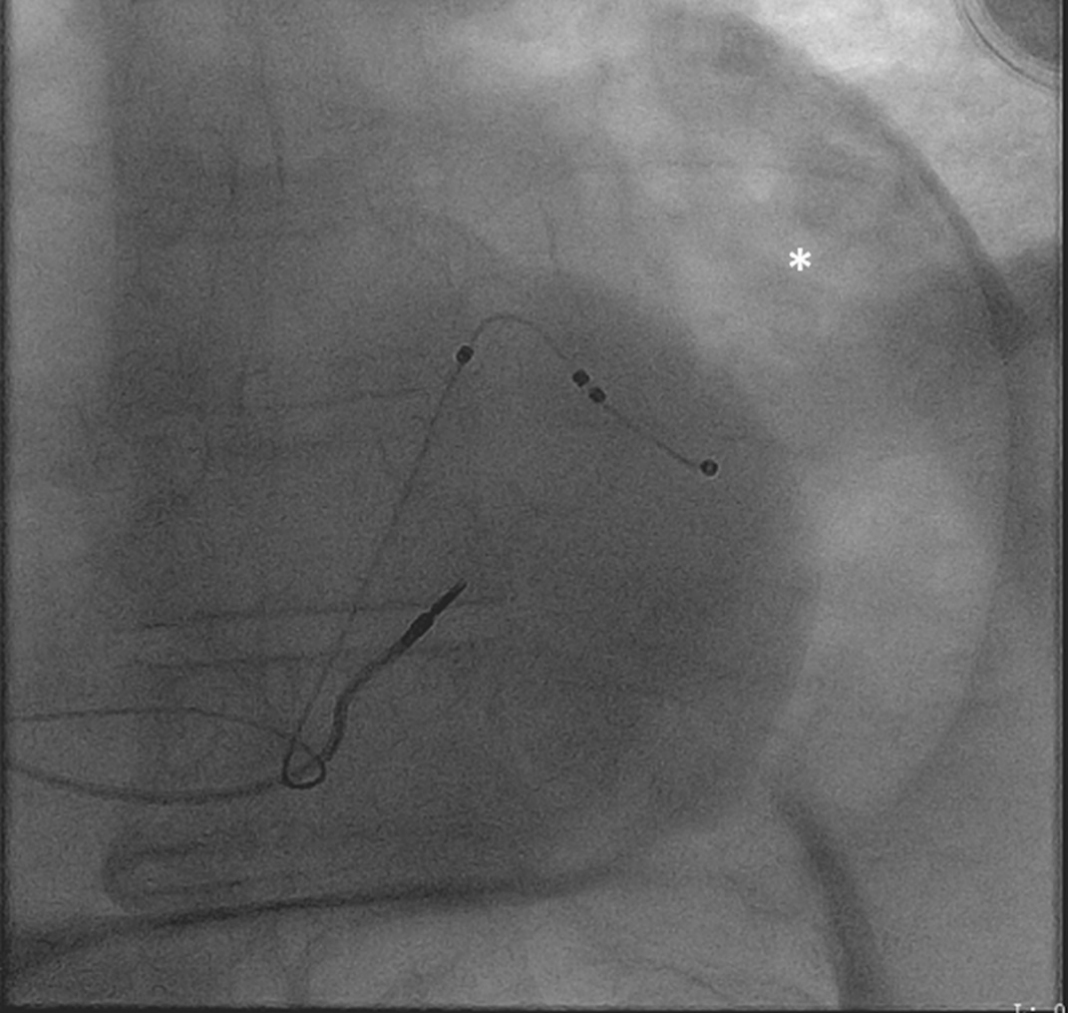
Pericardiocentesis Fluoroscopy showing evidence of a large pneumopericardium **(asterisk).**

**Figure 6:**
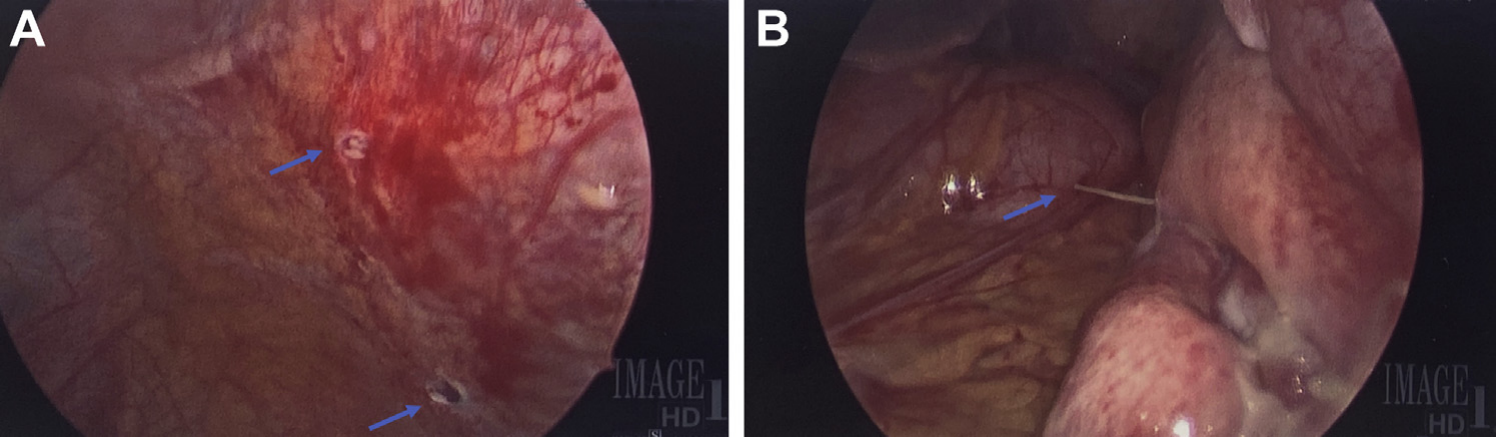
Repair of Puncture Sites **(A)** Laparoscopy revealing 2 diaphragm puncture sites **(arrows). (B)** Laparoscopic view of a colonic puncture site **(arrow).**

Pericardiocentesis was performed using the subxiphoid approach under echocardiographic guidance; 1.2 L of straw-colored fluid was removed, and a pigtail pericardial drain was left in place. Pericardial fluid analysis showed an elevated red blood cell count of 5,245/mL and a white blood cell count of 744/mL. Given the presence of subtle diffuse ST-segment elevations after pericardiocentesis, it was believed that the pericardial effusion was caused by underlying pericarditis. The drain was removed in 2 days when minimal drainage was noted. Immediately after removal of the drain, an audible hissing sound was heard. The site was immediately covered with petroleum gauze dressing out of concern for entrainment of air into the pericardial sac. An emergency chest radiograph revealed pneumopericardium and pneumoperitoneum (Figure 3). These findings were confirmed on computed tomography (CT) (Figures 4A and 4B). He remained hemodynamically stable. Pericardiocentesis was performed (Figure 5), with removal of 300 mL of air and 210 mL of serosanguineous fluid. The pericardial drain was left in place and attached to a Pleur-Evac system (Teleflex). Subsequently, the patient was taken to the operating room, where 2 diaphragm puncture sites (Figure 6A) and a transverse colon puncture were identified (Figure 6B); these puncture sites were repaired. Subsequently, pericardial fluid cultures grew *E coli,* which was attributed to contamination of the pericardial space secondary to perforation of the bowel by the pericardiocentesis needle before entering the pericardial space. An intravenous combination of ampicillin and sulbactam was initiated on the basis of sensitivity results, and there was no evidence of bacteremia. The pericardial drain was removed after several days; however, subsequent imaging showed reaccumulation of pericardial effusion, for which a pericardial window was performed. Results of pericardial fluid cytology and pericardial biopsy were nonrevealing. The patient was discharged home in stable condition with uneventful outpatient follow-up.

## Discussion

Pneumopericardium is an accumulation of gas, typically air, in the pericardial cavity. It is a rare, life-threatening condition that most often results from blunt thoracic trauma and can potentially lead to tension pneumopericardium (1). Infrequently, this complication can also occur after pericardiocentesis (2). Rarely, pericardiocentesis can lead to colonic perforation and pneumoperitoneum in the setting of a primary bowel disease (3). Pericarditis from an enteric organism has been reported as a rare complication of hepatic abscess (4), but there are no reported cases associated with pericardiocentesis.

An initial diagnosis of pneumopericardium can be made on the basis of a chest radiograph or CT scan (5). Once the diagnosis is established, management depends on the hemodynamic status of the patient. Although the development of pneumopericardium is an unfavorable prognostic marker (6), stable patients can be managed by treating the underlying condition and with supportive care with close monitoring (7). However, in the case of hemodynamic compromise, urgent pericardiocentesis is indicated (6). On rare occasions, when pneumoperitoneum occurs in the setting of pericardiocentesis, it can also be potentially managed conservatively (3), given the small diameter of the pericardiocentesis needle. Management of simultaneous pneumopericardium and pneumoperitoneum is even more ambiguous because there has been only 1 reported case of this unique disorder (8), which occurred in the setting of surgical pericardiotomy.

In our case, the patient had significant bowel distention in the setting of chronic constipation, in addition to an elevated left hemidiaphragm (Figure 1). This increased the risk of pericardiocentesis through a subxiphoid approach. Although immediate concern after hearing the whooshing sound was entrainment of air into the pericardial sac from outside, the sound was caused by the pressure filling of the peritoneum and pericardium by the air escaped from the hollow viscus. Pericardiocentesis using the subxiphoid approach led to transverse colon perforation. An alternative parasternal approach to pericardiocentesis would have likely prevented this complication. The patient did not manifest any symptoms of bowel perforation, pneumopericardium, or pneumoperitoneum until the drain was removed. The perforations were likely sealed by the pericardial drain; once the drain was removed, the condition manifested shortly afterward. Growth of *E coli* in the pericardial fluid confirmed contamination by an enteric microbe. Given his symptoms and early signs of shock, emergency pericardiocentesis was performed, followed by laparoscopic repair of the punctured bowel with an eventual need for a pericardial window.

## Follow-Up

At 1-month follow-up, there was no evidence of reaccumulation of pericardial effusion or evidence of pneumopericardium.

## Conclusions

This is an exceedingly rare complication of pericardiocentesis in the setting of bowel distention. This patient was successfully treated with multispecialty care. Knowledge of such complications is helpful in treatment planning and clinical approach to care.

## Funding Support and Author Disclosures

The authors have reported that they have no relationships relevant to the contents of this paper to disclose.
